# Silymarin Ameliorates Diabetes-Induced Proangiogenic Response in Brain Endothelial Cells through a GSK-3*β* Inhibition-Induced Reduction of VEGF Release

**DOI:** 10.1155/2017/2537216

**Published:** 2017-10-25

**Authors:** Ahmed Alhusban, Enaam Alkhazaleh, Tamam El-Elimat

**Affiliations:** ^1^Clinical Pharmacy Department, College of Pharmacy, Jordan University of Science and Technology, Irbid 22110, Jordan; ^2^Medicinal Chemistry & Pharmacognosy Department, College of Pharmacy, Jordan University of Science and Technology, Irbid 22110, Jordan

## Abstract

Diabetes mellitus (DM) is a major risk factor for cardiovascular disease. Additionally, it was found to induce a dysfunctional angiogenic response in the brain that was attributed to oxidative stress. Milk thistle seed extract (silymarin) has potent antioxidant properties, though its potential use in ameliorating diabetes-induced aberrant brain angiogenesis is unknown. Glycogen synthase kinase-3*β* is a regulator of angiogenesis that is upregulated by diabetes. Its involvement in diabetes-induced angiogenesis is unknown. To evaluate the potential of silymarin to ameliorate diabetes-induced aberrant angiogenesis, human brain endothelial cells (HBEC-5i) were treated with 50 *μ*g/mL advanced glycation end (AGE) products in the presence or absence of silymarin (50, 100 *μ*M). The angiogenic potential of HBEC-5i was evaluated in terms of migration and *in vitro* tube formation capacities. The involvement of GSK-3*β* was also evaluated. AGE significantly increased the migration and tube formation rates of HBEC-5i by about onefold (*p* = 0.0001). Silymarin reduced AGE-induced migration in a dose-dependent manner where 50 *μ*M reduced migration by about 50%, whereas the 100 *μ*M completely inhibited AGE-induced migration. Similarly, silymarin 50 *μ*g/mL blunted AGE-induced tube formation (*p* = 0.001). This effect was mediated through a GSK-3*β*-dependent inhibition of VEGF release. In conclusion, silymarin inhibits AGE-induced aberrant angiogenesis in a GSK-3*β*-mediated inhibition of VEGF release.

## 1. Introduction

Diabetes mellitus (DM) is a wide-spread chronic metabolic disease characterized by elevated blood glucose either due to insulin resistance (type II) or insulin deficiency (type I) [1–3]. Around 8.5% of adults aged 18 and older had elevated blood glucose in 2014 [4]. The prevalence of diabetes is increasing over the world and the expected number of patients to be diagnosed with DM is estimated to reach 366 million by the year 2030 [5].

Diabetes mellitus is a well-known major risk factor for the development of cardiovascular disease, including coronary artery disease (CAD), stroke, and peripheral artery disease [1]. About 80% of deaths among diabetics are due to atherosclerosis-related diseases [6, 7]. The majority of diabetes-associated complications are caused by its effect on both small and large blood vessels. Additionally, diabetes was found to alter angiogenesis in a tissue-dependent manner [8].

Angiogenesis is a multistep process in which new blood vessels are formed from preexisting ones [9]. This process is highly regulated by a balance between pro- and antiangiogenic factors to meet the metabolic demand of the body [10]. Angiogenesis has been shown to play an essential role in many physiological conditions, such as wound healing and growth [11]. Brain angiogenesis is involved in a multitude of brain functions, including learning [12] and recovery after ischemic insults [13–16]. Additionally, the endothelial cells of the brain have been found to release a number of growth factors, including vascular endothelial growth factor (VEGF), brain-derived neurotrophic factor (BDNF), and stromal-derived factor (SDF) that has been shown to be involved in recovery after CNS ischemic insults [17].

Diabetes has been found to impair angiogenesis in the peripheral vascular beds [6, 7, 18]. In contrast, it was found to have a proangiogenic response in the brain and retina [6, 8, 19–21]. In the brain, diabetes-induced proangiogenic response is characterized by generation of immature and fragile blood vessels [8]. This dysfunctional angiogenic response has been linked to a higher incidence of hemorrhagic transformation [8]. Prakash et al., 2012, demonstrated that diabetes-induced altered angiogenic response is mediated through oxidative stress-induced VEGF expression and release [8]. This finding suggests a potential role of antioxidants in ameliorating diabetes-induced dysfunctional angiogenesis.

Silymarin, extracted from the dry seeds of milk thistle [*Silybum marianum* (L.) Gaertn. (Asteraceae)], is a mixture of at least seven flavonolignans, silybin A, silybin B, isosilybin A, isosilybin B, silychristin, isosilychristin, and silydianin, and the flavonoid taxifolin [22]. It has been used for hundreds of years for liver diseases such as liver cirrhosis and chronic hepatitis [23]; clinical studies confirmed its hepatoprotective properties [24].

Silymarin has been widely studied for its profound biological activities particularly in cancer chemoprevention and hepatoprotection areas [25–27]. The biological effects of silymarin were also assessed in areas such as Alzheimer's disease [28], Parkinson's disease [29], and diabetes [30].

Silymarin with its potent antioxidant activity, low toxicity, and cellular protective effects compared with placebo encourages the exploration of its therapeutic potential uses [24].

Gadad et al., 2013, demonstrated the ability of silymarin to overcome diabetes-induced reduction in the migration of human dermal microvascular cells [31]. It is still unknown whether silymarin would have the ability to counteract diabetes-induced proangiogenic response in the brain given the tissue-dependent effect of diabetes on angiogenesis.

Recently, diabetes has been found to increase the expression and activity of glycogen synthase kinase-3*β* (GSK-3*β*). GSK-3*β* in turn has been found to play a significant role in regulating the expression of angiogenic factors such as VEGF and the angiogenic process in endothelial cells [32]. It is still unknown whether antioxidants like silymarin would alter the activity of GSK in endothelial cells or not. Accordingly, this study aims at evaluating the inhibitory potential of silymarin on diabetes-induced aberrant angiogenesis.

## 2. Materials and Methods

### 2.1. Cell Culture

Human brain microvascular endothelial cell line HBEC-5i (ATCC® CRL-3245™) was purchased from ATCC (ATCC; Manassas, VA) and was cultured in DMEM:F12 purchased from Euroclone (Euroclone S.p.A; Pero, Italy) supplemented with microvascular endothelial cell growth kit-BBE (ATCC; Manassas, VA). The cells used in the study were p1-5.

### 2.2. Treatments

*Silybum marianum* seed extract (silymarin) was obtained from Indena S.p.A. (Milan, Italy) (lot number 27691). The extract was analyzed for its content of eight individual bioactive components using a validated UHPLC-tandem mass spectrometry method [33]. The extract content of taxifolin, isosilychristin, silychristin, silydianin, silybin A, silybin B, isosilybin A, and isosilybin B was found to be 4.13, 1.22, 11.60, 7.02, 10.29, 15.68, 4.92, 2.52%, respectively, which sum to a total analyte content of 57% [33].

To prepare the stock solution, silymarin was dissolved in 1 mL of DMSO that was further diluted with serum-free media to a final concentration of 12.5 *μ*M. Silymarin was used in two concentrations (50 and 100 *μ*M). Advanced glycation end (AGE) products were purchased from Tocris (Tocris; Minneapolis, MN) and were reconstituted with serum-free DMEM:F12 to a final concentration of 250 *μ*g/mL. The concentration used in the experiments was 50 *μ*g/mL according to published literature [34, 35]. GSK-3*β* inhibition was achieved using 10 nM of SB-216763 (Tocris; Minneapolis, MN) that was dissolved in 0.0001% DMSO. DMSO was purchased from Santa Cruz Biotechnology (Santa Cruz Biotechnology; Dallas, TX). The cells were treated with silymarin 30 min before AGE application, whereas SB-216763 was applied 30 min before silymarin application.

### 2.3. VEGF ELISA

The concentration of VEGF released into the media was measured using a commercially available ELISA kit purchased from Abcam (Abcam; Cambridge, MA) according to the manufacturer recommendations. Briefly, conditioned media was collected 16 h after the application of different treatments and centrifuged at 10,000 rpm for 10 min. The supernatant was isolated and stored in −60°C until the time of analysis.

### 2.4. Angiogenesis Assays

#### 2.4.1. Cell Migration

The migratory capacity of HBEC-5i was measured using the wound healing assay. Cells were cultured in a 12-well plate to about 80% confluence before being serum starved for 24 h. A scratch was introduced in the cell monolayer using a 1 mL pipette tip, and the media was replaced by fresh serum-free DMEM:F12 media. The different treatments were sequentially added according to the abovementioned order. Images of the scratch edges were captured using a digital camera mounted on an inverted microscope at baseline and 16 h after treatment. The migration rate was determined by measuring the distance between the scratch edges using National Institutes of Health ImageJ software at both time points. The migration rate was calculated by subtracting the scratch width at 16 h from the width measured at baseline and dividing it the width at baseline. The wound recovery rate was presented as a percentage of the recovery rate of the control.

#### 2.4.2. Tube Formation Assay

The tube formation potential of HBEC-5i was determined using the *in vitro* tube formation assay according to published literature [32]. Briefly, 20,000 cells/well were added to a Cultrex® basement membrane extract-coated 96-well plate. The plate was coated with Cultrex basement membrane extract purchased from Trevigen (Trevigen; Gaithersburg, MD). To coat the plates, basement membrane extract was allowed to thaw at 4°C for 24 h before the experiment and 50 *μ*L were added per well of a prechilled 96-well plate. The treatments were applied as mentioned above. The tube formation rate was measured by counting the number of tube-like structure at 6 and 8 h after treatment application in three nonoverlapping images of each well.

### 2.5. Statistical Analysis

All experiments were repeated at least three times in duplicates. Statistical significance was determined using one-way ANOVA followed by the post hoc Tukey test. All statistical analyses were carried out using GraphPad Prism version 6; GraphPad Software, La Jolla, California. All values were reported as mean ± standard deviation error of mean (SEM). Statistical significance was considered as *p* < 0.05.

## 3. Results

### 3.1. Silymarin Does Not Affect Endothelial Cell Migration Rate

**Figure 1:**
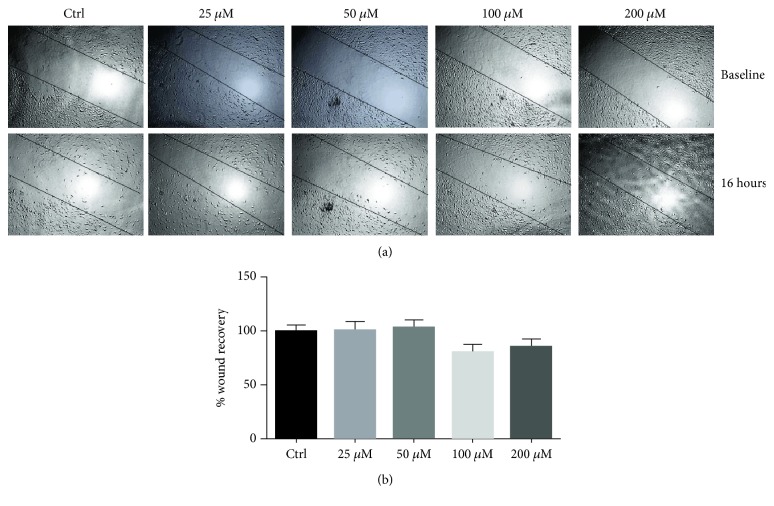
The effect of silymarin on the migratory potential of untreated HBEC-5i. Human brain endothelial cells were treated with a range of silymarin concentrations, and the migration rate was measured. Representative images of the migrated cells (a) and a quantification of the migratory potential (b). Silymarin did not alter the migration rate of endothelial cells. Data are presented as mean ± SEM; ∗ indicates a significant difference from control. *n* = 3.

To investigate the ability of silymarin to directly alter the angiogenic potential of untreated HBEC-5i, a range of silymarin concentrations (0–200 *μ*g/mL) was applied to HBEC-5i (Figures 1(a) and 1(b)). Silymarin in doses up to 200 *μ*g/mL did not have an appreciable effect on the migration of HBEC-5i when assessed 16 hours posttreatment.

### 3.2. Advanced Glycation End Products Increased the Migration and Tube Formation Rates in HBEC-5i in a Time-Dependent Manner

**Figure 2:**
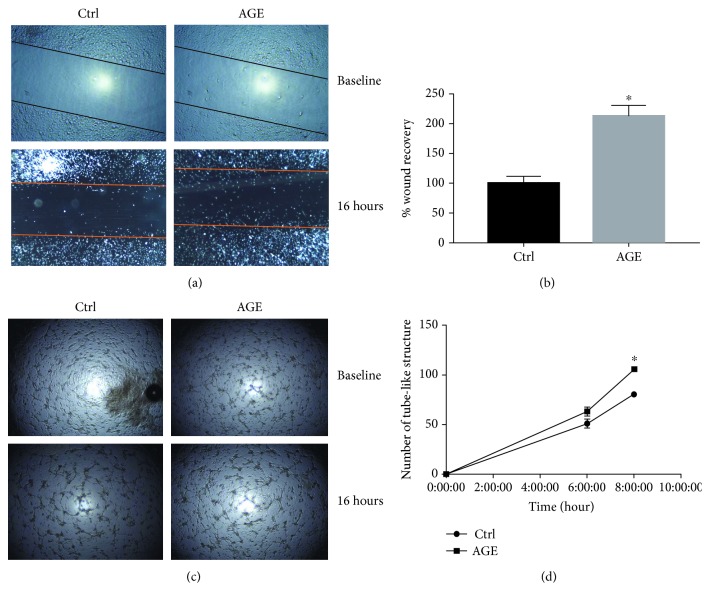
Advanced glycation end products induce a proangiogenic response in endothelial cells. Endothelial cells were treated with 50 *μ*g/mL of AGE, and the migration and tube formation rates of the cells were assessed. AGE increased the migration rate of endothelial cells as well as the rate of tube formation in a time-dependent manner. Representative images of the migrated cells (a) and a quantification of the migratory potential (b). Representative images of the tube formation potential of endothelial cells (c) and its quantification (d). ∗ indicates a significant difference from control. Data are presented as mean ± SEM. *n* = 3.

Diabetes has been found to increase the migration and tube formation capacity of brain endothelial cells [8]. Furthermore, the incidence of diabetes-induced complications has been found to increase with time [6, 18]. Accordingly, we assessed whether the effects of AGE would mimic the effects of diabetes on the behavior of HBEC-5i. AGE induced a onefold increase in the migratory rate of HBEC-5i (*p* < 0.0001) when assessed 16 hours posttreatment. (Figures 2(a) and 2(b)). Additionally, AGE induced a modest increase in the tube formation rate of HBEC-5i when measured at 6 h. Two hours later, AGE induced a 25% increase in the tube formation rate of endothelial cells (*p* < 0.05) (Figures 2(c) and 2(d)).

### 3.3. Silymarin Inhibited AGE-Induced Migration in a Dose-Dependent Manner

**Figure 3:**
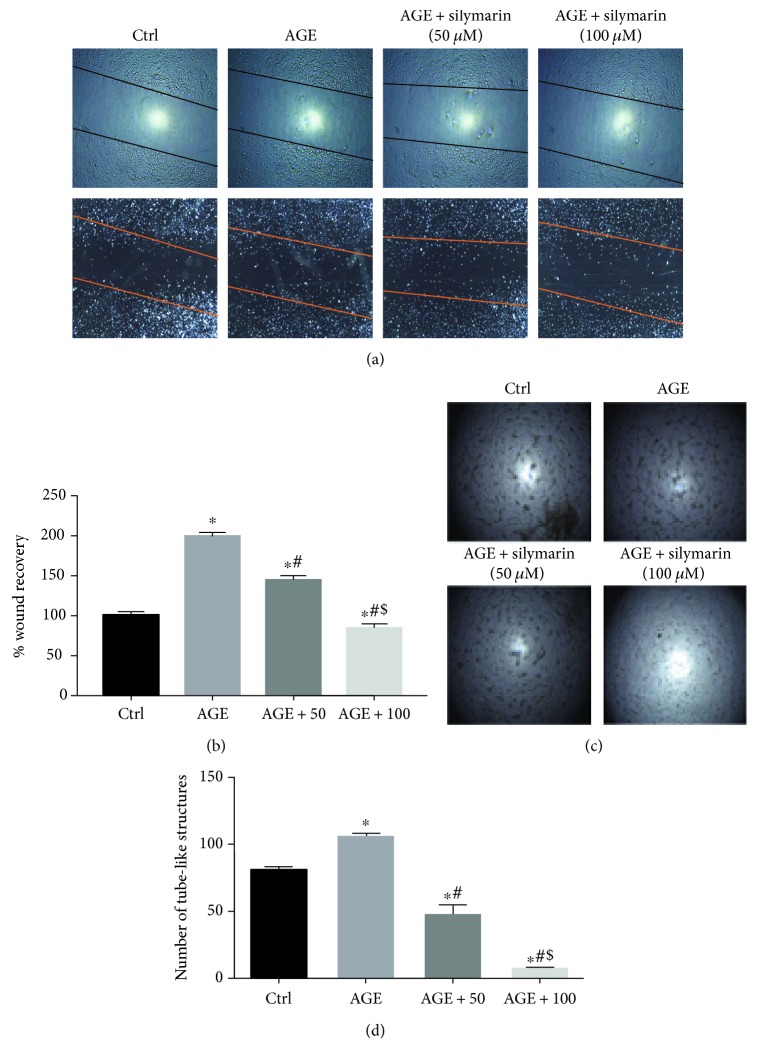
Silymarin inhibited diabetes-induced proangiogenic response in brain endothelial cells. Advanced glycation end products (50 *μ*g/mL) increased the migration rate of hBMECs. Treatment with silymarin inhibited AGE-induced brain endothelial cell migration in a dose-dependent manner. Representative images of the migrated cells (a) and a quantification of the migratory potential (b). Similarly, silymarin inhibited AGE-induced angiogenic response in human brain endothelial cells in a dose-dependent manner. Representative images of the tube formation potential of endothelial cells (c) and its quantification (d). Data are presented as mean ± SEM, *n* = 3–6. ^∗^*Significantly different* as compared to control; ^$^*significantly different* as compared to AGE; ^#^*significantly different* as compared to AGE + 50 *μ*g/mL-treated cells.

To assess the ability of silymarin to counteract AGE-induced effects in HBEC-5i, cells were treated with silymarin in two different doses (50 and 100 *μ*M). Silymarin at the 50 *μ*M concentration reduced AGE-induced HBEC-5i migration by about 50% when assessed 16 hours posttreatment (Figures 3(a) and 3(b)). Doubling the concentration of silymarin completely blunted AGE-induced migration when assessed 16 hours posttreatment (Figures 3(a) and 3(b)).

### 3.4. Silymarin Inhibited AGE-Induced Tube Formation in HBEC-5i in a Concentration-Dependent Manner

Silymarin at the 50 *μ*M concentration blunted the angiogenic potential of AGE-treated HBEC-5i (*p* < 0.001) (Figures 3(c) and 3(d)). Similarly, treatment with the higher concentration level of silymarin (100 *μ*g/mL) resulted in a more pronounced reduction of the tube formation rate in AGE-treated HBEC-5i (*p* < 0.001). The tube formation rate in the cells treated with 100 *μ*M was significantly lower than that in the AGE-treated group and the low-dose silymarin-treated group (Figures 3(c) and 3(d)). Interestingly, both concentrations of silymarin reduced the tube formation rate of AGE-treated HBEC-5i as compared to the control.

### 3.5. The Role of GSK-3*β*-Mediated Signaling in AGE-Induced Migration of HBEC-5i and Silymarin-Induced Amelioration of its Effects

**Figure 4:**
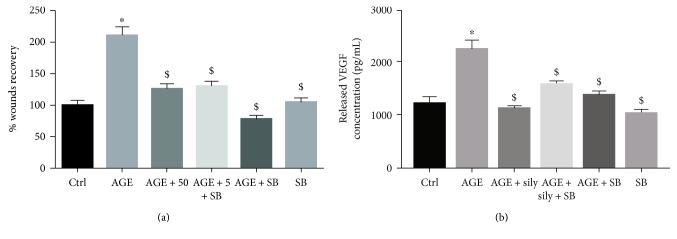
Silymarin inhibits diabetes-induced proangiogenic response in a GSK-3*β* inhibition-dependent reduction of VEGF release. Treatment with silymarin or GSK-3*β* inhibition using 10 nM of SB-216763 reduced AGE-induced migration in HBEC-5i. Cotreatment with both GSK inhibitor and silymarin resulted in a comparable inhibition to what is achieved in either of them (a). Treatment with AGE induced a onefold increase in VEGF release. This increase was blunted with silymarin cotreatment. GSK inhibition blunted AGE-induced VEGF release and did not alter silymarin-induced VEGF release inhibition (b). *n* = 3. ^∗^*Significantly different* as compared to control; ^$^*significantly different* as compared to AGE.

To investigate the involvement of GSK-3*β*-mediated signaling in AGE-induced effects in HBEC-5i and its amelioration by silymarin, the activity of GSK-3*β* was inhibited using the compound SB-216763 (10 nM) in AGE-treated cells. GSK-3*β* inhibitor blunted the AGE-induced migratory response in HBEC-5i when assessed 16 hours posttreatment (*p* < 0.05) (Figure 4(a)). The migratory response of untreated HBEC-5i was not affected by GSK-3*β* inhibition. Interestingly, GSK-3*β* inhibition in silymarin-treated HBEC-5i did not alter silymarin-induced effects (Figure 4(a)).

### 3.6. Silymarin Ameliorates AGE-Induced Angiogenic Response by Reducing VEGF Release

Treatment with AGE induced a onefold increase in VEGF release from endothelial cells (*p* < 0.05) (Figure 4(b)). Treatment with silymarin blunted the AGE-induced VEGF release (*p* < 0.05).

### 3.7. Silymarin-Induced Inhibitory Effect on VEGF Release May Be Associated with GSK-3*β* Inhibition

Glycogen synthase kinase-3*β* inhibition blunted AGE-induced VEGF release from HBEC-5i (*p* < 0.05) (Figure 4(b)). Similarly, silymarin inhibited AGE-induced VEGF release. Inhibiting GSK-3*β* in HBEC-5i treated with both silymarin and AGE did not alter VEGF release compared with AGE and silymarin cotreatment. This finding suggests the involvement of GSK-3*β* in silymarin-induced effects.

## 4. Discussion

The results obtained in this study demonstrated, for the first time, that silymarin ameliorates diabetes-induced angiogenesis in brain endothelial cells through a GSK-3*β*-mediated inhibition of VEGF release. Silymarin reduced both the migration and tube formation rate in HBEC-5i in AGE-treated cells. In contrast, silymarin did not have any appreciable effect in untreated cells except at very high doses. Furthermore, we demonstrated the essential role of GSK-3*β* in diabetes-induced dysfunctional angiogenesis. GSK-3*β* inhibition inhibited diabetes-induced migration and VEGF release.

In this investigation, we used advanced glycation end (AGE) products to model diabetes *in vitro*. There are plenty of methods to model diabetes *in vitro* [8, 34, 35]. Additionally, AGE and its receptor (RAGE) are implicated in a variety of pathological conditions that may not involve hyperglycemia [6, 9, 18, 34–42]. In contrast, AGE has been widely accepted as a substance that can induce the detrimental effects of diabetes and model diabetes *in vitro* [34, 35, 40]. Furthermore, the observed effects of AGE in endothelial cells are consistent with the reported effects of diabetes in the same type of cells [8]. Accordingly, we are confident that our model is valid and truly reflects the effects of diabetes in endothelial cells.

Data from experimental studies demonstrated the ability of diabetes to induce a proangiogenic state in the brain [8, 14]. This angiogenic response is characterized by the formation of nonperfused fragile blood vessels [8, 14–16]. Additionally, diabetes was found to impair the neuroprotective effects of brain endothelial cells [34, 35]. Collectively, these data demonstrate the detrimental effect of diabetes on brain endothelial cells. Additionally, they explain the larger infract, poor outcome, and impaired recovery after ischemic insults among diabetic individuals [14, 15, 43, 44]. Accordingly, ameliorating diabetes-induced angiogenesis would offer an intriguing target to prevent diabetes-induced aggravation of CNS ischemic insults.

Diabetes is associated with a high level of oxidative stress [19, 20, 45, 46]. Prakash et al., 2012, demonstrated the ability of antioxidants to inhibit diabetes-induced angiogenesis using FeTPPS [8]. This compound accelerates the degradation of peroxynitrite and thus reduces diabetes-induced oxidative stress [8, 47]. Unfortunately, this compound is highly toxic and [8, 47]. Additionally, it has been found that epicatechin can reduce diabetes-induced pathologic angiogenesis in the retina [19]. These data in addition to other highlight the potential of antioxidants to ameliorate diabetes-induced abnormal angiogenesis [8, 14–16, 19, 21, 43, 45, 46, 48, 49]. An essential prerequisite for the success and applicability of this approach is the use of safe antioxidants.

Silymarin is a natural antioxidant with a long history of safety and efficacy [23, 24, 26, 30, 50]. Gadad et al., 2013, tested the ability of silymarin wafers to inhibit diabetes-induced impairment of human dermal microvascular cell migration [31]. Although interesting, their work focused on the migratory capacity of human dermal microvascular endothelial cells. Additionally, they did not characterize the molecular pathway of silymarin-induced effect. Furthermore, the type of the cells they used is essential in wound recovery after peripheral injuries but cannot extrapolate to endothelial cells from other vascular beds. Accordingly, assessing the ability of silymarin to ameliorate diabetes-induced angiogenesis and the involved molecular pathway is of utmost importance to establish the potential use of silymarin in preventing diabetes-induced dysfunctional angiogenesis in the brain.

In this investigation, we tested the hypothesis that silymarin ameliorates diabetes-induced abnormal angiogenesis in the brain in a GSK-3*β*-mediated inhibition of VEGF release. Similar to what has been reported by Gadad et al., 2013, silymarin inhibited diabetes-induced alteration in endothelial cell migration [31]. In contrast, to what has been reported in human dermal microvascular cells, silymarin reduced diabetes-induced migration in brain endothelial cells in a dose-dependent manner. This discrepancy is mainly related to the differential effect of diabetes on microvascular endothelial cells [8]. Prakash et al., 2012, reported a proangiogenic effect of diabetes on endothelial cells of the brain and retina [8]. In contrast, endothelial cells from peripheral vascular beds exhibit an impaired migratory potential in response to diabetes [8]. This impaired migratory potential is the main cause of impaired wound healing in diabetic patients [8]. Accordingly, silymarin normalizes the endothelial cell response to diabetes.

In this investigation, we expanded the work reported by Gadad et al., 2013 [31], by assessing the ability of silymarin to ameliorate diabetes-induced alteration in the ability of endothelial cells to form tube-like structures. Our data demonstrated, for the first time, that silymarin reduces diabetes-induced tube formation potential of endothelial cells. Interestingly, both concentrations of silymarin reduced the capacity of endothelial cells to less than that observed in untreated endothelial cells. In contrast, the low dose of silymarin (50 *μ*g/mL) reduced the migration of AGE-treated endothelial cells by about 75% as compared to untreated endothelial cells. The high concentration of silymarin completely blunted AGE-induced migration and reduced it to the level detected in the untreated cells. A similar differential effect on the migration and tube formation in brain endothelial cells has been reported previously in response to angiotensin II [32]. Alhusban et al., 2013, reported that 1 *μ*M of angiotensin II reduced the angiogenic potential of brain endothelial cells while the migratory capacity was not affected by the same concentration [32].

Diabetes was found to increase VEGF expression and release in primary brain microvascular endothelial cells [8]. Furthermore, treatment with an anti-VEGF prevented diabetes-induced angiogenesis [8]. Similarly, diabetes increased VEGF release in the human brain microvascular endothelial cell line used in this study. Silymarin reduced diabetes-induced VEGF release. Accordingly, silymarin-induced normalization of the angiogenic response in HBEC-5i is mediated through silymarin-induced inhibition of VEGF release. This finding highlights the potential utility of silymarin as an intervention to reduce VEGF release. It is still unknown whether this effect is mediated through a reduction of VEGF expression or an inhibitory effect on VEGF secretion from HBEC-5i.

Glycogen synthase kinase-3*β* (GSK-3*β*) has been shown to function as a signaling node in the brain [51]. It integrates signals from outside and inside the cell and modulates the expression of growth factors such as VEGF [51]. Furthermore, it modulates the activity of intracellular adhesion molecules [51]. Accordingly, GSK-3*β* has an essential role in angiogenesis, neurogenesis, and recovery after CNS ischemic insults [13, 51]. Diabetes was found to increase GSK-3*β* activity [52, 53]. It is still unknown whether GSK-3*β* is involved in diabetes-induced dysfunctional angiogenesis. Our results showed that GSK-3*β* inhibition antagonizes diabetes-induced migration of HBEC-5i. This finding highlights a potential role of GSK-3*β* in diabetes-induced dysfunctional angiogenesis. Additionally, inhibiting GSK-3*β* in silymarin-treated cells did not alter silymarin-induced inhibition of diabetes-induced migration. This finding suggests that silymarin-induced effects may be induced through GSK-3*β* inhibition. Confirming this finding requires assessing the phosphorylation level of GSK-3*β* in silymarin-treated cells and comparing it to that in silymarin-untreated cells using western blotting. Currently, we are in the process of conducting this investigation.

## 5. Conclusion

Silymarin inhibits AGE-induced brain angiogenesis in a dose-dependent manner. This inhibitory effect is induced through a GSK-3*β*-mediated inhibition of VEGF release. Additionally, we demonstrated the potential role of GSK-3*β* in diabetes-induced abnormal angiogenesis.
